# The primary care experience of adults with chronic obstructive pulmonary disease (COPD). An interpretative phenomenological inquiry

**DOI:** 10.1371/journal.pone.0287518

**Published:** 2023-06-23

**Authors:** Sanduni Madawala, Narelle Warren, Christian Osadnik, Chris Barton

**Affiliations:** 1 Department of General Practice, School of Public Health and Preventive Medicine, Faculty of Medicine, Nursing and Health Sciences, Monash University, Prahran, Victoria, Australia; 2 Department of Sociology, School of Social Sciences, Faculty of Arts, Monash University, Prahran, Victoria, Australia; 3 Department of Physiotherapy, School of Primary and Allied Health Care, Faculty of Medicine, Nursing and Health Sciences, Monash University, Prahran, Victoria, Australia; The University of Manchester Division of Psychology and Mental Health, UNITED KINGDOM

## Abstract

**Background:**

Studies of the lived experience of chronic obstructive pulmonary disease (COPD) reveal a number of challenges patients face when interacting with healthcare providers that may be exacerbated by unwillingness or inability to quit smoking. However, none have explored, in-depth, primary care experiences among patients with COPD in community healthcare settings.

**Aims/ objective:**

The study investigated healthcare experiences of patients living independently in the community with COPD who smoked or had recently quit (at most within the last 5 years), seeking care in primary care settings.

**Method:**

An Interpretative Phenomenological Analysis (IPA) involving thirteen participants purposively recruited from social media posts in COPD and carer support groups, general community groups, community noticeboards and paid adverts on social media. In-depth interviews were held between February and April 2022 by phone or Zoom^™^ and explored patient experience of primary care, focusing on how smoking patterns, addiction and stigma impact upon and shape these experiences.

**Results:**

Participants were aged between 45 to 75 years. Nine were female and two thirds were current smokers. Problematic experiences including time-constrained consultations, having to self-advocate for care *“…go digging myself and then go and see him and say*, *can we do this*, *can we do that type of thing*?” and guilt about smoking were common. Positive care experiences described non-judgemental interpersonal interactions with doctors, timely referral, proactive care and trust “*I have an actual great trust for my GP… they’re awesome*, *they’ll look after you*”. Participants described how their care experience shifted as primary care adapted care delivery during COVID-19.

**Conclusions:**

Pro-active, empathetic care from general practitioners is desired from patients living with COPD. Stigma and fear of judgement was an important underlying driver of negative care experiences contributing to delayed help seeking from general practitioners.

## Introduction

Chronic obstructive pulmonary disease (COPD) is a preventable, progressive respiratory illness and major contributor to global morbidity and mortality [1–4]. Smoking remains a major risk factor for developing COPD. In such people, smoking cessation is the single most important treatment goal to slow disease progression [5, 6]. Prevalence is disproportionately higher among individuals in the lowest compared to highest socioeconomic groups [7, 8], mirroring rates of smoking in the community [1]. The negative effects of COPD on daily activities [9] result in substantial social impacts for individuals [10]. Central to effective management of COPD, particularly for mild to moderate severity, is high quality care coordinated by community-based general practitioners (GPs) [11] focusing on areas such as optimising symptoms [12], reducing risk factors (e.g. smoking) [11, 13, 14], improving quality of life [15, 16], and managing complex issues following acute exacerbations [17].

Achieving effective engagement between patients and health care providers, however, is not without challenge [14, 18–21]. Barriers related to stigma, lack of motivation, poor quality of life and guilt among ongoing smokers contribute to such complexity [14, 18, 20–22]. Feelings of judgement contributing to guilt and shame has also reportedly contributed to patients hiding their diagnosis [23]. Concerns such as these were exacerbated during public health responses to the COVID-19 pandemic [24].

The ‘patient experience of care’ describes various interactions and relationships formed between patients and different components of the health care system, including family practice. It comprises both structural elements of care like waiting times, consultation structure, and the physical environment of the clinic as well as affective, or relational, components such as respect, communication and trust [25]. Wong and Haggerty describe six essential dimensions to thoroughly understand the patient experience in primary healthcare [26]. These are access, interpersonal communication, continuity and coordination, comprehensiveness of services, trust and patient-reported impacts.

One element of the care experience of particular relevance in the context of smoking related disease is the influence of stigma [19]. The experience of stigma has been found to act as a barrier to accessing and appropriately engaging with healthcare providers in studies exploring insights of current and ex-smokers across countries including Australia [19, 20], Sweden [27], Ireland [28] and Norway [29]. Boland’s study [20] found Australian smokers from low SES backgrounds perceived stigmatising attitudes when accessing treatment, and shame associated with previous failed attempts at quitting smoking and failure to overcome the addiction. Similar to this, those with COPD report experiences of stigma in healthcare settings which were found to exacerbate poor patient experience of care [22].

Qualitative studies exploring the lived experiences of people with COPD suggest discordance between patients’ expectations of support and the support they actually receive [19]. A poor patient experience of care can result in sub-optimal treatment and outcomes, consequently exacerbating one’s experience (i.e. ‘real’) or threat (i.e. ‘anticipation’) of stigma. This is a particular risk for people with COPD who continue to smoke, however knowledge regarding this group is limited.

This study aimed to describe the experiences of primary healthcare among a group of adults with COPD who report a history of smoking. We sought to better understand the significance of smoking in these participants’ lives and how perceptions of stigma shaped COPD patients’ experiences of care.

## Methods

### Study design

This study was guided by interpretative phenomenological analysis (IPA) [30–33]. The latest version of IPA was used [31]. This method allows in-depth exploration and insight to how participants make sense of their personal and social ‘world’ or surroundings. It provides understanding of experiences, events and states reported by participants [31, 32] and is well suited to exploring topics requiring detailed, in-depth focus on an individual’s experience [32] and those that are complex or ambiguous [33].

#### Positioning of the researcher

Awareness of the researcher’s position in relation to participants (i.e. the insider-outsider perspective) is important in phenomenology due to their potential to influence all steps of the research process [34]. In this study, participants were persons living independently in the community, who had been told by their doctor they have COPD, and who had been unable to, or unwilling to, cease smoking after their diagnosis. The researcher (SM) was a non-smoker who possessed a Psychology degree and was undertaking a PhD. The researcher had no direct experience of airways disease, either personally or in the care of others, and approached patients and data analysis as an empathetic outsider; not a clinician researcher or someone with personal experience living with or caring for people with chronic airways disease.

Reflexivity is also central to phenomenological research and the IPA method [35]. Guided by experienced mentors, SM undertook a process of reflexivity as the primary investigator and author, seeking to engage in a ‘self-understanding’ process about her bias, values and experiences [35] in order to identify the ways in which these experiences may have shaped interpretation of participants’ experiences. Strategies to achieve this included keeping a reflexive journal, involvement of a multi-disciplinary team in the project and identifying researcher perspectives, positions, values and beliefs throughout the process.

### Participants and sampling

#### Setting

Participants were Australian residents living independently in the community with a self-reported doctor-diagnosis of COPD. They required at least one GP visit for their COPD in the past year. While not a criteria for eligibility, the presence of comorbid chronic illnesses, both physical and mental, were common.

#### Sampling

IPA studies require relatively small, demographically homogenous samples that include key attributes that potentially influence the phenomena of interest. Purposively sampling was conducted using i) social media posts in COPD and carer support groups, general community groups, community noticeboards and paid adverts on social media; ii) study promotion via paper advertisements in various community organisations and community settings (e.g. community centres, community bulletin boards, libraries); and iii) advertisement in a local council publication in south east Melbourne. Participants who responded to the Facebook advertisement and met eligibility criteria were contacted by the researcher (SM) through email and phone to schedule an interview time. Explanatory statements and consent forms were emailed to participants following this and consent was obtained prior to the interview through email or mail. Socio-demographic information were collected via electronic form in Qualtrics (Provo, UT) prior to the initial interview. A small subsample of past smokers were also interviewed to gain further insights and allow comparison with current smokers, and to explore divergent experiences [33] related to quitting smoking. All participants who had quit smoking reported quitting within the past 5 years.

### Data collection

In-depth interviews, guided by a semi-structured interview guide, were conducted remotely via telephone or by Zoom^™^ due to ongoing COVID-19 public health measures at the time. All interviews were conducted by SM, and took place between February and June 2022. Interviews ranged from 23 minutes to 163 minutes, spread over two sessions approximately 1 month apart. The follow-up interview was guided by questions formed based on important topics that were raised in the initial interview and required further follow up. This helped the researcher gather rich information, while minimising participant fatigue which is common in COPD [36]. The interview guide (available in online supplement, see S1 Appendix) was developed in line with recommended principles [31, 32, 37]. It comprised a series of broad topics to guide discussions including health status and main concerns, smoking behaviours and patterns, experience of care (guided by Wong and Haggerty’s framework [26]), smoking cessation, and experiences of stigma in healthcare settings. Topics were presented in an unstructured manner (i.e. precise wording and order determined by participant engagement and interview flow), and probing questions and prompts were developed to facilitate in-depth responses, where needed.

The interview guide was ‘pilot tested’ with the first three participants. These interviews were transcribed verbatim and discussed among the authors to refine the questions and ensure the scope and focus of the interview captured relevant detail, and to refine the student’s interview technique. These pilot interviews were deemed to be of sufficient standard to be retained for analysis. Audio recordings of all subsequent interviews were transcribed verbatim by a professional transcriptionist as soon after interviews as practical. Short field notes were made during the interview and a more detailed, reflective summary was completed by the interviewer (SM) in her research journal after each interview to supplement recorded data. Result summaries were made available for participant feedback prior to analysis (see S1 Fig).

### Data analysis

QSR NVIVO version 12 was used to support data management during the coding and analytic process which was led by the primary author (SM). Regular meetings were held with the authorship team to discuss emerging interpretations and understanding of the participants experiences. Interpretations and understanding of meanings in data were challenged and shaped areas for clarification or exploration in subsequent interviews with participants and later analytic work.

Reflections from the research journal influenced data collection and analysis. Firstly, reflections from the research journal were used to develop questions for the follow-up interview. Similarly, patient experiences issues that were highlighted by participants as having a major negative impact on their experience of care were recorded in the research journal and these were closely analysed.

Analysis was guided by recommendation of Smith and Nizza [31]. The iterative process comprised four distinct stages.

1. Thorough reading and re-reading of transcripts and the recording of descriptive and conceptual comments [31, 32]. These included summaries, paraphrases, associations, connections and preliminary interpretations. Both descriptive and conceptual comments were made to create these initial exploratory notes describing interactions with the health system, health staff and ultimately making sense of health care experiences in the broader context of each participants lives. Once complete for all participants, NVivo was used for further coding and to organise codes into preliminary themes.
2. Forming case summaries for each participant, informed by key quotes and the interviewer’s initial interpretations, notes and experiential statements. Similar statements and codes were arranged into groups, as themes began to emerge. In this context, themes were higher-level expressions encapsulating the concepts within a set of codes.
3. Theoretical ordering of group experiential themes. This highly analytical phase involved extraction of exemplar quotes from each transcript and cross-checks against original transcripts (primary source) and audio to ensure contextual accuracy.
4. Cross-case analyses, involving identification of similar experiential statements across participants and structuring these under ‘group’ experiential themes. These were discussed between two authors extensively (SM and CB) and a final set of group experiential themes were selected and named that encapsulated the experience of this group of participants.

Lincoln and Guba’s evaluative criteria were applied to achieve rigour and trustworthiness in the collection and analysis of data. These included credibility, transferability, dependability and confirmability [38]. Interpretative phenomenological analysis is embedded in the constructivist paradigm. Constructivism is linked to the key epistemological assumption that knowledge is constructed from perception and experience. Similarly, the corresponding ontological assumption posits that reality is based on human and social construction [35, 39]. Coding was guided by the patient experience domains identified by Wong and Haggerty’s framework [26]. However, considering IPA is primarily based on constructivist principles, coding was largely inductive.

### Ethical considerations

The study was approved by Monash University Human Research Ethics Committee (#27076). Participants provided written, informed consent prior to participation. Interviews were offered over two time points to manage issues of breathlessness or fatigue that might have impacted participants ability to participate. If participants became distressed during the interview, we offered to take a break, however none opted to withdraw and all interviews were completed without any issues of concern. Participants were able to withdraw from the study and request for their data to be removed, however none did.

## Results

Characteristics of the thirteen participants are presented in Table 1 (For detailed participant demographics see online supplement table in S1 Table). One participant completed just a single interview and did not respond to follow up requests. Two participants self-identified as being at Stage 4 COPD, others described their symptoms as mild or moderate. Most participants lived in the state of Victoria (n = 7), four lived in Queensland and one each lived in Western Australia and New South Wales. Four identified their gender as male, and nine females. Ten participants were Australian and three described themselves as New Zealander, but living in Australia. Ten participants were aged under 64 years, two were 65 to 74 years and the oldest participant was 75 years of age. They mainly had concerns about physical activity such as walking, breathing and coughing.

*“the main problem is that I feel walking is*, *it’s always been really difficult for me actually*. *I get short of breath very easily*. *So that’s that*, *and because I’m a smoker I sometimes cough in the morning*, *not all the time*.”

*[ID-10*, *female*, *55–64*, *Western Australia*]

*“I was having difficulty breathing because I think the humidity and the air is just heavier up here (Queensland) than it is down there (Victoria)*.*… when you have exacerbations that’s when it gets scary*, *that’s when you can’t breathe and you feel like*, *someone’s*, *described it as you feel like you’re drowning*, *which it’s just because you can’t get any air*. *It’s like you’re suffocating*.”

[ID-2, female, 55–64, Queensland]

**Table 1:** Summary of participant characteristics.

		N (%)
Age	45–54	5 (38.46%)
	55–64	5 (38.46%)
	65–74	2 (15.38%)
	75+	1 (7.69%)
Gender	Male	4 (30.77%)
	Female	9 (69.23%)
State of residence	Victoria	7 (53.85%)
	Queensland	4 (30.77%)
	NSW	1 (7.69%)
	Western Australia	1 (7.69%)
Modified Monash Model^1^	Metro (MM 1)	9 (69.23%)
	Regional centre (MM 2)	3 (23.08%)
	Rural (MM 5)	1 (7.69%)
Smoking status	Current smoker	
	• Daily	6 (46.15%)
	• At least once a week	1 (7.69%)
	• Less than weekly	1 (7.69%)
	Ex-smoker	5 (38.46%)
Education	University degree	3 (23.08%)
	TAFE/Diploma	3 (23.08%)
	Year 12	3 (23.08%)
	Finished prior to year 12	4 (30.77%)
Marital status	Married / De-factor	8 (61.54%)
	Divorced / widowed / single	5 (38.46%)
Employment status	Employed / self-employed	4 (30.77%)
	Unemployed / student/stay at home	5 (38.46%)
	Pension / retired	4 (30.77%)

^1^ Modified Monash Model: MM1-Metropolitan areas; MM2-Regional centres; MM3-5-Large, medium and small rural towns; MM6-7-very remote & remote communities [58]

**Fig 1:**
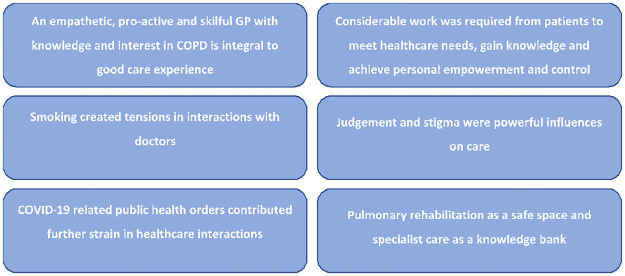
Summary of the six group experiential themes that described participants experiences of care.

Participants described a range of experiences when interacting with health services for their COPD and several ‘group experiential’ themes were identified. A series of six themes best represented the data and health care experiences of this group of patients. Each theme is presented below together with exemplar quotes from participants in Fig 1.

### An empathetic, pro-active and skilful GP with knowledge and interest in COPD is integral to a good care experience

A GP that was perceived as “doing all they could to address my health needs” was respected by participants. Having a GP that understood ‘the big picture’ was important and satisfaction with care occurred when their GP provided whole-person care, involved the participants in health-related decision making, was proactive and interested in the individual. A supportive GP helped the participant do what was best individually for them and encouraged social and physical activities. A GP with a non-judgemental attitude who was knowledgeable about when and where to refer patients to specialist and allied health services was valued. Being given accurate information about COPD and the services available to them was important to participants, and meant patients felt cared for and that their concerns were not brushed aside. Participants described feeling cared for and trusted GPs with these attributes.

*“I had always liked his non-judgmental manner*. *He was the one who first told me I had COPD*. *And you know he was always quick to refer on when needed*.”

*[ID-10*, *female*, *55–64*, *Western Australia*]

*“…I have an actual great trust for my GP… [they are] awesome doctors … and they actually really do*, *like these guys once you go in they start to pump you through a system of okay*, *you haven’t had this checked out and we’ve noticed that*, *so go and speak to this person here and they’ll—do you know what I mean*?”

[ID-4, male, 45–55, Victoria]

However, few participants could identify with a care provider like this and overall there was a call to action for GPs to keep up to date on COPD-related knowledge. Participants wanted GPs to be proactive and to involve them in care decisions and be clearer about when to seek follow-up care, and how to access allied health services (like pulmonary rehabilitation) that could help them. On the whole, participants commonly felt ‘confused’ and ‘disillusioned’ about their care and condition.

While some participants accepted the limitations of their GP’s knowledge and did not expect them to have specialist knowledge there was a clear need for more information about life expectancy, quality of life and the role and interplay of smoking on life expectancy. Participants expressed, simply, a need for more information about ‘what to expect’. Participants were less trusting and ‘talked’ less with a GP who was perceived as having little knowledge about COPD and little interest in obtaining the knowledge. This was particularly important to participants who lived with multiple health conditions.

Participants reported a variety of emotions and responses when receiving their diagnosis of COPD; some were not surprised at all while others were shocked at the stage of diagnosis, the manner in which the diagnosis was delivered and discouraging responses of healthcare professionals. It was important for health professionals to alleviate these concerns by providing information, talking to patients about the issue and having information packs available regarding what to expect with the condition.

*“it’s making me more determined to try and get the right answers because there’s now so much confusion and so much lack of knowledge*, *I sort of want to get knowledge and I think I’m entitled to it*.”

*[ID-3*, *male*, *65–74*, *Victoria*]

### Considerable work was required from patients to meet healthcare needs, gain knowledge and achieve personal empowerment and control

Participants wanted a GP that assisted them with the ‘work’ of managing COPD, that provided individualised treatments and made participants feel their health was taken seriously. Participants described having to do additional work to understand the condition and its treatments. A ‘proactive’ GP was characterised as someone who was good and caring.

*“… I think that makes you feel that you are*, *someone’s taking your health seriously … he’d [previous GP] looked outside the square as well and he’d go let’s look further at this and this and this*.”

*[ID-10*, *female*, *55–64*, *Western Australia*]

*“(my) GP pushes on issues*, *she doesn’t sweep things under the rug*, *she keeps asking and doing the work required of a GP*.”

*[ID-9*, *female*, *45–54*, *Victoria*]

Participants described the ‘work’ they needed to do to fill gaps left in their care. A lack of trust and lack of rapport, and sense of limited time within appointments, contributed to this sense of unmet need and frustration.

“I’m going to go and learn what I can from whoever I can and then take it from there. … I’ve been seeing him for over 12 months (but) he hasn’t taken the time to get to know me, because I don’t think he’s got the time.”

*[ID-6*, *female*, *55–64*, *Queensland*]

A lack of information from the GP was a recurring concern and some participants did not become aware of the seriousness of emphysema until later in the course of their illness.

*“I didn’t realise that emphysema was such a big thing*, *… my doctor prior to the one I’ve got now … was a smoker himself; so he was quite casual when he told me probably long before I was diagnosed with COPD and other problems*, *that I had mild emphysema … I’ve always been brought up thinking that emphysema is just a bad cough that’s going to get worse*.”

*[ID-3*, *male*, *65–74*, *Victoria*]

*“So it’s*, *it’s just learning more about it and that’s something that I never learnt from any GP*. *No GP has ever given me any written brochures*, *information*, *no one has ever said*, *look maybe you should go and see a specialist or anything*. *So it was just me asking to see a specialist and the information that I got from this pulmonary rehab which is great*.”

*[ID-6*, *female*, *55–64*, *Queensland*]

The difficulties experienced by GP’s to meet patients’ needs were attributed by participants to an over-burdened health system and this was also seen in the difficulty experienced by some participants, particularly those in regional and rural areas, in finding timely appointments. Further to issues of access, coordination of care between providers and between services often did not meet participants needs or expectations? Participants described coping as best as possible until an appointment was available despite worsening symptoms.

*“my original doctor … he could always fit you in*. *If you needed to see him that day he would see you that day*, *that was never a problem*. *He would always sit down there and discuss everything with me and what the outcomes are going to be and everything else … (my new doctor) she’s so over loaded with work she loses track of where she even is and I think this is a lot of the problem*, *I think everyone is over worked*, *the whole system is over worked*. *I think (that’s) what is causing the problem*. *… it’s the whole system (at fault)*”

*[ID-3*, *male*, *65–74*, *Victoria*]

*“My old GP had lots of programs and things*, *like they had mental health help and support*, *and they had a phlebotomist in their GP that you could get bloods done straight away*, *so you didn’t have to go somewhere else*. *They had a really good nurse that was in touch with lots of different things and could link you up with lots of different things*. *This one*, *there’s none of that*, *I don’t know if it’s because they’re new and they’re still building the practice or what it is*, *but yeah there’s none of that*.”

*[ID-5*, *female*, *45–54*, *Victoria*]

Limited availability of consultation times impacted the patient experience across a range of domains, including accessing care, interpersonal communication and their interpersonal relationship with the GP. Spending time with patients and feeling heard, with empathy and compassion, were key factors participants desired in the doctor-patient relationship. Continuity of care and being able to be seen regularly by a GP was important to all participants but difficulties accessing appointments and limited time within appointments meant they were unable to raise important health concerns.

*“And so that’s continuity*, *and development in—or relationship with your GPs it’s huge*. *I hate being forced to see somebody else if some—or if mine’s not available*. *And I—I … time*, *keep them to a minimum*. *I’m—I lower my expectations*. *And I sometimes will even avoid broaching topics*.”

*[ID-9*, *female*, *45–54*, *Victoria*]

*“Oh god yes*, *get it all over and done with and sorted out in one hit*. *You’ve got seven minutes*, *no longer*, *no shorter—seven minutes to get everything out of the way*. *If it’s not done he taps his watch and says make another appointment*.”

*[ID-12*, *female*, *55–64*, *rural Queensland*]

*“Yeah overall I would say I was satisfied with him*, *but I just think it’s the*, *it’s the way things are these days*, *you just can’t have a family doctor because it’s*, *they don’t have the time and to spend with you the way they used to which is a shame*, *which is a real shame*.”

*[ID-6*, *female*, *55–64*, *Queensland*]

Limited follow-up from GP’s left some participants with a lack of confidence in the healthcare they received. Some were able to advocate for themselves and initiate appropriate follow up, but others struggled with this and felt ‘uncared’ for. This contributed to a sense of nihilism from some participants who were reluctant to access treatments or additional tests if they felt the GP was not willing to help. For example, participant 6 described how they will *“…go digging myself and then go and see him and say*, *can we do this*, *can we do that type of thing*?”. In contrast, participant 3 felt that “…*if you’re not getting to the bottom of it and if you haven’t got the person who wants to get to the bottom of it what’s the point in doing another scan*?”

Amongst this group there was a strong desire for more proactive care, but many felt they were left with too much of the ‘work’ required for their care.

Several participants felt their GP’s were dismissive of their health concerns. They felt like a number and disrespected. These participants were less trusting of their GP’s and made comments such as “*they’re only good for a prescription and a referral*” [ID-1, female, 45–54, Victoria].

*“Yeah*, *particularly when I felt so bad and that doctor turned around and said just deal with it*, *it’s emphysema*, *that happens; because I said I don’t understand how it happened overnight like that and he said well it can and it does*, *so just deal with it*.”

*[ID-3*, *male*, *65–74*, *Victoria*]

*“…yeah I’m just a number I’m not a person*. *You’re barely even looking at me*, *you wouldn’t be able to recall what I looked like at the end of your day*, *because you’re just looking down*, *looking at your computer screen*.”

*[ID-10*, *female*, *55–64*, *Western Australia*]

### Smoking created tensions in interactions with doctors

Most participants reported beginning smoking around the age of 15 or 16 years and reported having no real ‘internal rationale or justification’ for having the urge to smoke. Anxiety, alcohol-use, habit, taking a break from work and passing time were triggers for smoking on a day to day basis. Many of the participants reported a significant event in the past, such as parents’ divorcing, or observing parents smoking that prompted them to begin smoking. One participant reported that therapy for quitting smoking unnecessarily probed into her childhood trauma, making her more depressed, feel inferior and ultimately leading her to stop attending counselling. She reported preferring to focus on the present and moving on from these events.

*“I’ve been to therapy*, *and I said*, *all they do is they always start with my upbringing*, *my childhood*, *my …*. *I had lots of things happen that weren’t good in my life*. *I blocked out half*, *probably from the first 10 or 12 years of my life … the memories were too bad*. *They … always goes [sic] back to that—you know*, *we have to go through that first*. *So*, *once I end up totally depressed and re-living it again—I quit*, *I won’t go*.”

[ID-11, female, 75–84, Queensland]

One participant who had not smoked for over 2 and half years described how they used ‘mental willpower’ to stop smoking and refers to it as ‘mental warfare’. Having smaller goals and overcoming them was an effective strategy for this participant and ‘hating’ the drug (tobacco) and its consequences appeared to be the first step, for them, to quit. However, one of the major difficulties he highlighted was quitting cigarettes appeared to be a silent killer, where the consequences are not obvious.

“*So and it was like for me to actually give up smoking was a mental warfare in its own right…And that’s one of the things that we’ve got to remember with smoking*, *is we always give ourselves permission … if we ask ourselves do we give ourselves permission then we might find that we say no and start saying no to ourselves on it because it’s very much cigarettes are a mental (thing)*”

[ID-4, male, 45–55, Victoria]

Receiving a diagnosis of COPD prompted some participants to reduce smoking, in some cases from thirty cigarettes per day to thirty over two weeks [e.g. ID-7, female, 65–74, Victoria]. A small number of participants who had recently quit smoking spoke about the financial burden of smoking, and the need to quit to reduce the impact on their health and wellbeing. One participant felt a focus on the smoking may have helped him quit. Importantly, he referred to himself as a ‘reformed’ smoker, highlighting that it is important to disclose current or past smoking to the doctor to receive appropriate care. Others felt it was important to them that their GP accepted their smoking without judgement, even if they continued to smoke. This involved discussing smoking cessation, discussing and prescribing smoking cessation aids for patients with COPD and giving the patient the time to prepare to quit. Participants did not want to be ‘pushed’ into stopping smoking and preferred feeling they were ready to quit smoking before having the conversation with their GP. Participants had a ‘common-sense’ understanding that their doctor would ask them about smoking and if they were ready to quit smoking. But some recounted accounts of ‘rude’, ‘impersonal’, ‘forceful’ comments that impacted their experiences of care. Examples of quotes from participants illustrating their perspectives on smoking cessation discussions are presented in Table 2.

**Table 2:** Quotes from COPD patients illustrating their perspectives on smoking cessation discussions with their GP.

ID	Quote
ID-3 male, 65–74, Victoria	*“Rather than ignore the fact and say no*, *I don’t smoke; I don’t smoke*, *that’s the end of it—but let them sort of try and work out something else*. *Maybe I should keep quiet but I don’t think that’s fair and I don’t think it’s doing me any good to say that either… That’s why I say I think there should be three sections*, *are you a smoker*, *a non-smoker or an ex-smoker; really important I think”*
ID-10 female, 55–64, Western Australia	*“He knew I was a smoker*, *… talking to me every time I went there about not smoking was not going to have an effect*. *So*, *he accepted me and he looked at ways that could help me as an individual*, *not as a standard this is what I have to tell everybody*. *Whereas I feel I haven’t built that relationship with my new GP yet*.*”*
ID-10 female, 55–64, Western Australia	*“I feel that from both GPs I’ve had*, *they’ve both imparted the information should I want to give up*. *They both made me aware of different things I could do to help*.*”*
ID-10, female, 55–64, Western Australia	*“If we go back to my past GP*, *he knew that I would get to that place if and when I was ready to*, *so no amount of pushing was going to change*. *So*, *it was always every appointment*, *still smoking*? *Yes*. *Ready to quit*? *No*. *Okay and left it like that*, *you know*. *I’ve said I’m not ready to quit*, *so why would he then start pushing*? *So that was good*. *Yeah*, *no*, *I don’t think pushing anyone into something they’re not ready for is going to be beneficial*.*”*
ID-5 female, 45–54, Victoria	*“No*, *when I went to the specialist … at [name] Hospital*, *I don’t think it was unfair*, *it was just very blunt*, *that you have to give up smoking*, *like yeah*, *but I don’t think it was unfair or anything*.*”*

Some participants reported being able to quit unassisted, however receiving encouraging advice from their GP was essential.

*“Well I’ve nearly quit*. *I’ve cut right*, *right*, *right back to next to nothing*, *that’s because of all the difficulties I’ve had up here with my breathing and walking and everything*. *But I’ve done all that on my own and with the help from my previous doctor*. *It’s always judged if you mention that you smoke*. *He hasn’t brought it up for a couple of months because he knows I’ve been dealing with lots of major other things at the moment*, *but he does know I’m trying to give up again*, *and he always encourages me*, *and says to keep on trying*, *never quit quitting type thing*.”

[ID-5, female, 45–54, Victoria]

Language that had a focus on positive change and empowerment was preferred to support quit attempts. While community standards and public health restrictions were cited as important in helping people quit, participants emphasised the importance of having respectful language where people who smoke are not labelled and treated like “lepers” [ID 3, male, 65–74, Victoria].

### Judgement and stigma were powerful influences on care

Many participants described stigmatising experiences and language from their GP. These experiences had impact upon help-seeking in the future.

*“Well because everything’s so hard to access*, *if you mention that you are still smoking*, *everybody doesn’t seem to take you seriously or as important*.”

*[ID-5*, *female*, *45–54*, *Victoria*]

One participant who had quit smoking (ID-6) described feeling how they are *“… only happy to go to GPs now because … I don’t smoke anymore*. *Whereas before I was so reluctant because I was being judged and it shouldn’t be that way*.” [ID-6, female, 55–64, Queensland]. She described the impact of feeling judged on her willingness to seek care as a ‘vicious cycle’ where she continued smoking, her symptoms worsened yet she continued avoiding going to the doctors for fear and anticipation of judgement. She described uncertainty and emotions of guilt due to her smoking, and judgement from health professionals. The ‘brutal’ comments from her doctor were not conducive to change.

*“you’re (unclear) made to feel guilty because you’re a smoker*. *I’ve done it to myself I know that*. *But I was at a point where I just wouldn’t go to the doctors unless I was half dead because they made you feel so bad…So I got another GP and he was an older (doctor) and he was the one that pretty much said to me*, *you’ll be lucky to see 65 if you keep going*. *And he was so brutal that I went back to work and they had to send me home*, *I didn’t get out of bed for 2 days … And I’m still sitting here thinking*, *well I’m 62*, *am I going to make it to 65*? *…So that was very brutal*. *And but he was probably hoping to scare the crapper out of me*, *but all it did was make me go home and smoke more*.”

*[ID-6*, *female*, *55–64*, *Queensland*]

Feelings of guilt and shame prevented this participant from seeking and learning important information about COPD and accessing additional community services such as pulmonary rehabilitation.

*“And yes I probably still go in sometimes scared*, *just probably from … from when I get frightened you* *probably went in*, *thinking they’d have a go at me again*. *What’s going to happen*? *Not that this doctor has actually ever given me a hard time*, *it’s just so many have given me a hard time that you do go in with a bit of negative feeling I think*. *But that’s probably internal in myself*.”

*[ID-6*, *female*, *55–64*, *Queensland*]

Receiving continuous care was important considering different doctors had “*different ideas who made you feel even worse because you were smoking and you had COPD*”, placing patients at risk of a re-experiencing stigma and resulting in a “love/hate relationship”

*[ID-6*, *female*, *55–64*, *Queensland*].

Not receiving important services such as social care, pulmonary rehabilitation, dietician, National Disability Insurance Scheme (NDIS) home-based services and specialist lung services (and other specialist care services such as oncology, etc) was disappointing to patients, especially if they observed others with a condition other than COPD had access to these services. This left participants with the perception that they were not receiving services because of COPD being a ‘self-inflicted condition’ and that their physical difficulties were not taken seriously.

*“Yeah and the fact that the lady next door to me has a brain tumour and so she has vision [problems] … she’s only 48 this month but she has so much help …–my unit and hers are both exactly the same*. *She even got a 3*,*400 aircon put in by the NDIS and shoes*, *she gets shoes*. *All I ever wanted was a blooming Social Worker*. *She has Social Workers*, *she has Vision Australia do just about everything for her … and like I don’t begrudge her any of that help but here’s me in the other unit gets*, *can’t even get a Social Worker*.”

[ID-11, female, 75–84, Queensland]

*“Moving out here the way I have*, *I’ve been quite emotional anyway*, *so*, *and I don’t know*, *some people just don’t seem to understand it or the impact of it all*. *That’s mainly*, *my job agency constantly on my back pushing me to get work*, *that they’re not taking all my health issues into consideration*, *and making me go for jobs that I just can’t physically do*, *yeah and just totally ignoring my (multiple) health issues*.”

*[ID-5*, *female*, *45–54*, *Victoria*]

### COVID-19 related public health orders contributed further strain in healthcare interactions

The COVID-19 pandemic, and the public health orders in Australia at different times to control its spread, added further strain to the difficult experience of care already encountered by these participants. Accessing a GP appointment appeared to be easier for some, but specialist appointments were difficult to access in some states. Additionally, COVID-19 had a significant impact on access to (in-person) pulmonary rehabilitation classes and exercising was difficult.

*“what has probably not helped is COVID*, *which is out of everybody’s control; but like I said*, *this girl [name]*, *she works at [name] Hospital*, *she started taking me through some exercises etc*., *and that’s had to come to a stop*. *We did speak on the phone not too long ago*, *but I’m pretty hopeless when it comes to … following up on what people say I suppose*, *I’m one of those people that sort of needs someone behind me whipping me with a strap or something saying c’mon*, *exercise*.” ’

*[ID-3*, *male*, *65–74*, *Victoria*]

*“sometimes when I had to have telephone appointments and there was something that he had to physically do or something than I could go in*. *Accessing any sort of specialists couldn’t be done…With the respiratory people again*, *but they weren’t taking people because it was inside the hospital and they weren’t letting people in*, *and I wasn’t dying*.”

*[ID-5*, *female*, *45–54*, *Victoria*]

In some cases, the GP was rated highly, however, the practice was rated low due to stigmatising experiences and inconvenient processes that were introduced, such as closing off waiting rooms, to minimise the spread of COVID-19. One participant described how she sought a mask exemption from their GP and then experienced distressing stigma from not wearing a mask in different healthcare settings such as the hospital waiting room. She described how “you’re the only one unmasked and you’re getting dirty looks from all the other masked patients.” *[ID-10*, *female*, *55–64*, *Western Australia]*. Some patients experienced difficulties engaging with medical receptionists that made them think “*It’s just not even worth going in*” (ID-10). GPs tended to be more understanding of the participant’s health and need for a mask exemption, and having access to tele-health appointments alleviated some of these concerns.

*“So she was coming from a caring*, *… place with her beliefs [the GP]*, *whereas yeah*, *the receptionist yeah*, *she definitely*, *there was a lot of stigma around that*.”

*[ID-10*, *female*, *55–64*, *Western Australia*]

Physical difficulties caused by the use of plastic barriers and difficulty with hearing through these barriers and masks added to the difficulty experienced by participants interacting with receptionists. In this context, using an online website for making appointments, which were introduced by many clinics in Australia during this period, was described as a useful tool to get an appointment rather than ringing or trying to set up an appointment face to face with confrontational interactions with practice reception staff.

### Pulmonary rehabilitation as a safe space and specialist care as a knowledge bank

Most participants who had attended pulmonary rehabilitation reported feeling emotionally safe and wished they had gone earlier. Pulmonary rehabilitation physiotherapists and nursing staff were important for raising awareness about services and encouraging patients to access them when needed. One participant had observed that pulmonary rehabilitation physiotherapists didn’t make those who were still smoking feel guilty during the program.

*“And but then there were still people in my course (pulmonary rehabilitation course) that were still*, *still smoked*. *And stuff like that but they didn’t*, *they didn’t give them a hard time about it*, *and make them feel guilty*”

*[ID-6*, *female*, *55–64*, *Queensland*]

*“Learning more about COPD and that*, *well someone said about a rehab*, *the pulmonary and that’s what made me Google it*… *maybe because I was feeling so guilty and judged that I didn’t go looking before but then the doctor should have*, *I feel like my doctor should have said*, *look maybe we should send you to rehab … I just*, *wish I would have done it earlier*,”

*[ID-6*, *female*, *55–64*, *Queensland*]

Pulmonary rehabilitation physiotherapists provided positive reinforcement and encouragement to participants while lung specialists gave COPD-specific advice to place the condition into perspective. However, some participants reported difficult stigmatising comments and responses from specialists.

*“I went to a specialist at the hospital a couple of weeks ago and he’s gone above and beyond what I expected*. *He’s been very*, *very thorough and taken the time to get all the information*, *to get all the bloods you know*.”

*[ID-10*, *female*, *55–64*, *Western Australia*]

*“my GP and my specialist (current)*, *because I trust them*. *It’s taken me*, *like five years to get them to be where I feel comfortable with them*. *But yeah*, *with other specialists I still just feel like they’re laughing behind my back*.”

*[ID-13*, *female*, *45–54*, *New South Wales*]

## Discussion

This study describes detailed primary healthcare experiences from a small group of adults with mild to moderate COPD living in the Australian community. Our study drew upon a broad conceptualisation of patient experience, and across these domains’ participants’ stories around smoking and the ‘tensions’ they encountered in their interactions with doctors could be described across six ‘group experiential’ themes. Experiences of stigma and judgement were a feature of interactions with doctors and health services that impacted all areas of the healthcare experience. Fear to access services and seek care from healthcare professionals arose from ‘felt’ and ‘experienced’ stigma. Participants identified traits such as empathy, pro-activeness and skill as important traits for GPs to possess. When they did not find these traits, they described high ‘work’ associated with self-management. They attributed short appointment times and constantly changing doctors to ‘changing times’ and ‘the current state of the healthcare system’. The difficulty felt in changing health providers meant participants had to accept a lower level of care than they felt they should—and that their status in the health system as ‘a smoker’ contributed to lower quality in service delivery than others were perceived to receive. In some cases, the reasons for smoking were complex and in one person, related to traumatic past events which posed challenges to adequately support decisions to quit. Many had unanswered questions about their health and management of their breathing problems which impacted upon their experience of care, and satisfaction with health care providers.

Past survey research has demonstrated that adults with COPD (current smokers and past smokers) were likely to ‘anticipate’ experiencing stigma in primary care more than past or current smokers with other chronic conditions. This impacts upon help-seeking and is strongly related to delayed or avoided care when needed [22]. This is contrasted with patient experience findings among the general Australian population which is typically rated as very high. A majority of people (98%) report having a regular GP and most (84%) describe receiving excellent or very good care [40]. The Australian Bureau of Statistics Patient Experience survey (2019–2020) [41] describes high access to services and consistently positive experiences among patients within the Australian general practice setting, although these numbers have been noted to decline since the emergence of the COVID-19 pandemic. Objective positive experience measures can, however, mask nuanced experiences [42]. International data from the USA and UK suggest positive patient experiences are closely associated with clinical effectiveness and patient safety [43]. Poorer patient experiences are observed to be more prevalent among those in lower socio-economic groups in Canada [44, 45]. This emphasises the importance of detailed exploration of patients’ experience of care and seeking understanding amongst vulnerable groups at risk of poor experiences of care. In-depth interpretative phenomenological analysis methods are highly suitable for this purpose [31].

The Australian experience of general practice, at a population level, is typically a positive one. It is concerning as such, that this group of particularly vulnerable patients, who are at risk of poor health outcomes, experience interactions with doctors and health services that don’t meet their expectations for care. Our findings are broadly consistent with international literature of COPD patient experiences of health care [19]. Underpinning these experiences are feelings of judgement and experiences of stigma, whether actually encountered or perceived.

The theoretical construction of stigma and understanding of its role in lived experience of illness has evolved over time. Erving Goffman’s seminal work (1963) [46] conceptualised stigma as a phenomenon that occurs as ‘society establishes the means of categorising persons and the complement of attributes felt to be ordinary and natural for members of each of these categories’. However, several theoretical frameworks have since emerged that build on this classical construct to provide a basis for exploring stigma in the context of healthcare experiences. In particular, the concept of ‘anticipated’ stigma has recently been developed by Earnshaw and Quinn [47], and describes the belief that prejudice, discrimination and stereotyping will be directed at the self from others *in the future*. This is a particular concern for individuals living with chronic illnesses [47–49] and is a concept that may underpin many of the barriers to effective health care reported in studies of smokers and other health conditions where there is societal stigma. This concept has been described in the care experience of patients with other stigmatised lung conditions such as lung cancer [50, 51] and also HIV [52] and diabetes [53]. The slow, progressive nature of the disease trajectory in COPD and the long term nature of the care relationship required for the patient with COPD is not dissimilar to the need for long-term care relationships in these other chronic conditions and lessons from the experiences of participants in our study may also benefit the care of other patient groups in general practice.

Patients require a tailored approach, from a trusted, knowledgeable clinician who will not judge them for their actions or use stigmatising or threatening language. Interestingly, group pulmonary rehabilitation provides a setting in which participants consistently reported positive health care experiences and should be offered to all general practice patients as part of their COPD care. Participants in our study described pulmonary rehabilitation as a safe space to learn about their condition and how to manage it. Other literature supports these findings and highlight how pulmonary rehabilitation is an important component in managing COPD [19, 54, 55], with evidence that it improves exercise capacity and health-related quality of life [54]. It is well known, however, that many patients who would benefit from pulmonary rehabilitation, and other allied health services, struggle to access them.

The concept of ‘work’ emerged from our interviews and described the additional, self-directed work that patients had to pursue to learn about their illness and its treatment. Considerable work was required from patients to meet healthcare needs, gain knowledge and achieve personal empowerment and control. Past research has described a high degree of illness burden amongst COPD patients including fatigue, difficulty with physical activity and cognitive deficits in adults with COPD [12, 56]. Our findings formally link these concepts to the experience of care and highlight the importance of providing *pro-active* support and care. Participants in our study with positive care experiences described their GP as “doing all they could to address my health needs”, understanding ‘the big picture’, involving patients in health-related decision making, being proactive and interested in the individual. Participants described feeling cared for, and trusted GPs with these attributes.

### Strengths and limitations

This is one of the most comprehensive, qualitative investigations of patient experience among this patient group, who experience a high degree of illness-related and social vulnerability due to smoking. The interviews were conducted by SM who was an *outsider*, in the sense of her relationship to study participants, but also as a non-clinician researcher. Careful consideration and reflection were required in the conduct and analysis of data in these contexts. However, her ‘outsider’ positioning was also a point of strength, as she was able to develop good rapport with her participants who were able to share their stories and felt safe in conveying their experiences of care.

While COVID-19 public health orders posed a challenge to achieve immersion in the lived worlds of these participants, efforts were taken to address this. Conducting an initial and then a follow-up interview resulted in over 30 hours of audio data and allowed the researchers time to explore and understand experiences important to each participant. It provided opportunity to probe key concepts raised in the initial interviews, and was an important part of the member checking process. In addition to conducting a second round of interviews, SM also joined online COPD support groups and observed a hospital-based Community Rehabilitation Centre to understand patient interactions with health providers. Discussion of disconfirming evidence and alternative explanations occurred at regular supervisory team meetings, and enabled inter-disciplinary perspectives to be incorporated into the findings.

Participants were emailed a summary of the study’s preliminary findings as a means to check data interpretations and supported credibility of findings. In the reporting of results, the experiences and impact are described richly (thick description) and supported by participants quotes and examples. External auditing by supervisors, who probed and challenged emerging explanations and understandings, assisted in maintaining dependability of data analyses and interpretations. Findings were presented to academic GPs and clinicians at seminars and conferences to gain external perspectives on preliminary findings.

Finally, we encountered both positive and negative experiences allowing us to compare and contrast what underpinned these differences. While our focus was current smokers, we also interviewed a small number of ex-smokers who had recently quit smoking, in order to gain those perspectives and evidence of change in care experience based on smoking status.

A limitation of the present research is that participants self-reported their diagnosis and severity of COPD, which we were unable to confirm. COPD is often poorly understood and many patients, especially with early signs of illness, may have been eligible to participate but would be unaware of their condition. Further, we relied on social media and recruiting participants from illness and carer support groups. Respondents from these channels may be inherently pro-active to engage in health care via such mediums, or conversely, have greater support needs than other patients in general practice.

## Conclusion

Participants described a range of experiences when interacting with health services for management of their COPD. Stigma was a powerful underlying driver of care experience for this group, that exhibited overt and covert impacts on health-seeking behaviours and other aspects of the healthcare experience. Perceptions of medical and social vulnerability were magnified as a result of COVID19 and stigmatising experiences encountered in health services from practice reception staff. Many described a heightened sense of ‘work’ associated with their care, which particularly impacted the experience of comprehensiveness and coordination of care. Proactive, empathetic care was important to participants but this was not the experience of all participants. The results of this study highlight the negative impact of stigma and indicate the need for multi-level interventions designed to reduce stigma [57]. Experiences of stigma reported in this paper also indicate a need for change within GP practices to deliver care that does not invoke feelings of guilt, shame and judgement for people with COPD.

## Supporting information

S1 Appendix Online supplement—Detailed interview guide. (DOCX)

S1 Table Online supplement—Detailed participant demographics. (DOCX)

S1 Fig Result summaries provided to participants for feedback. (PDF)
