# Supplementary material for: The primary care experience of adults with chronic obstructive pulmonary disease (COPD). An interpretative phenomenological inquiry
Source: PLoS One. 2023 Jun 23;18(6):e0287518. doi: 10.1371/journal.pone.0287518 (PMC10289323; doi:10.1371/journal.pone.0287518)
Supplement: S1 Appendix — (DOCX) [file pone.0287518.s001.docx]

**S1 Appendix. Online Supplement - Detailed Interview Guide**

Topic area 1: Health status and main concerns

Prompt question: Can you tell me a little bit about your breathing problems, what are the main concerns you have with your breathing at the moment?

Prompt question: So, thinking back to when you were first diagnosed with COPD, can you tell me about what the GP told you and how you felt?

Topic area 2: Smoking behaviours and patterns

Aims: To understand the significance (impact) of smoking in participants' lives and how COPD patients who have not quit smoking construct a justification for smoking.

Prompt question: Tell me about when you started smoking?

Probe: How patterns have changed over time

Probe: Current smoking amounts

Prompt question**:** Are there things that you enjoy about smoking?

Probe: Do have any Triggers for smoking

Probe: Explore justification for ongoing smoking / internal rationalisation: Can you tell me a bit about how you justified the smoking?

Prompt question: Have you thought about quitting smoking? Tell me about the last time you tried to quit smoking.

Probe: Explore starting and stopping, and why they re-started / recidivism / relapse

Probe: Was this self-initiated or did your regular GP initiate?

Prompt question: Tell me about your experiences related to feeling addicted/the urge to smoke?

Probe: Addiction

Topic area 3: Experience of care

Aims: Understand how patients with COPD who smoke conceptualise good quality care from their GP. What do they expect from their primary care providers?

Prompt question: Let’s talk about your GP and doctors that you see. Tell me about your GP and the clinic that you go to most often

Probe/Prompt: Regular GP? How long going to this clinic?

Probe/Prompt: Access Issues

Probe/Prompt: Continuity of care and coordination?

Probe/Prompt: Comprehensiveness of service at practice

Probe/Prompt: Relational and Affective elements / interpersonal, the relationship with your GP

Probe/Prompt: Trust

Probe/Prompt: Overall satisfaction/rating of GP care

Prompt question: How have you felt about going to the doctors during the COVID Pandemic

Topic area 4: Primary health care and smoking cessation

Aim: Do patients perceive GP's to be a key point of contact/ key external support in quitting smoking

What barriers do COPD patients face in accessing smoking cessation support from their primary care provider?

Prompt question: When was the last time that you talked to your GP about smoking and quitting smoking?

Probe: what did they recommend (advice_

Probe: What was useful advice, what advice was not useful?

Probe: How do you feel when your GP brings up the topic of smoking?

Probe: Did you find they helped in the manner you expected?

Prompt question: Did they suggest any treatments or refer you to others to help

Probe: Pharmacological / Behavioural / Counselling

Prompt question: Readiness to engage with your healthcare provider to quit smoking

e.g. Can you tell me how ready you are to work with your GP to quit smoking?

Prompt question: Do you see your GP as a key point of contact and external support in quitting smoking?

Topic area 5: Stigma in primary healthcare setting

What are experiences of stigma in primary care settings, and how have these shaped interaction with healthcare providers (in the context of smoking cessation).

How does the anticipation of stigma, or fear of being judged about smoking, shape interactions with GPs and access to primary care?

Prompt question: Have you experienced feeling judged about your smoking? Can you explain

Probe: fear of being judged in the primary care setting? Has this impacted the way you interact with GP’s and accessing primary care?

Probe: Anticipation of this kind of judgement when interacting with GP’s? Has this impacted the way you interact with GP’s and accessing primary care?

Prompt question: Experiences/Enacted of stigma in health care settings e.g. Have you experienced unfair treatment with a healthcare provider or in a healthcare setting? Do you think it was because of the smoking?

Probe: Examples of behaviours / language etc. experienced that induced feelings of stigma

**Internalised stigma**

Probe/Prompt – explore how internalised

Prompt question: Have you experienced any thoughts or emotions that may come from feeling judged in the primary care or other health care settings?

Prompt question: How do those feelings/incidences that you describe make you feel about going to the doctor the next time

Probe– how impacts upon perception of accessibility

Probe –interaction with health care providers

Probe – how they view their doctor – interpersonal relationship

Probe: Societal stigma

Prompt question – What are your experiences of community standards about smoking, do they impact you?

Probe: Do you find these community standards help you to quit smoking or do they act as barriers?

Probe: Do these community standards impact upon your interactions with healthcare, with the GP or any other healthcare setting?

Topic area 6: Other Healthcare settings

Prompt question: Experiences with healthcare providers in different settings

Probe/Prompt: Pulmonary rehabilitation

Probe/Prompt: Specialist care/outpatient setting

Probe/Prompt: Behavioural Counselling/ Psychologist

Probe/Prompt: Smoking Cessation services

Probe/Prompt: Social Work/care

Probe/Prompt: Dietician/Nutrition
