# Supplementary material for: The primary care experience of adults with chronic obstructive pulmonary disease (COPD). An interpretative phenomenological inquiry
Source: PLoS One. 2023 Jun 23;18(6):e0287518. doi: 10.1371/journal.pone.0287518 (PMC10289323; doi:10.1371/journal.pone.0287518)
Supplement: S1 Table — (DOCX) [file pone.0287518.s002.docx]

**S1 Table. Online Supplement – Detailed participant demographics**

|  | State of residence | How often do you **now** smoke cigarettes, pipes or other tobacco products | How long ago did you quit smoking daily? | mMRC* | In the past 12 months, have you been to see a GP for your own health? | What is your gender? | What is your age? | With which ethnic group do you identify? | What is the highest degree or level of school you have completed? | What is your marital status? | What is your current employment status? |
| --- | --- | --- | --- | --- | --- | --- | --- | --- | --- | --- | --- |
| ID-1 | Victoria | Daily | Within last year | 0 | Yes | Woman | 45-54 | Australian | Completed year 12 | Defacto | Employed |
| ID-2 | Queensland | Less than weekly | Within last year | 0 | Yes | Woman | 55-64 | Australian | Completed more than 3-year university degree | Divorced | Out of work and looking for work |
| ID-3 | Victoria | Not at all but I have been a regular smoker | 3-5 years | 2 | Yes | Man | 65-74 | Australian | Completed Year 12 | Married | Retired |
| ID-4 | Victoria | Not at all but I have been a regular smoker | 3-5 years | 1 | Yes | Man | 45-54 | Australian | Completed Year 12 | Single | Student |
| ID-5 | Victoria | Daily |  | 1 | Yes | Woman | 45-54 | Australian | TAFE/diploma graduate | Widowed | Out of work and looking for work |
| ID-6 | Queensland | Not at all but I have been a regular smoker | Within last year | 1 | Yes | Woman | 55-64 | Australian | Some high school, but did not complete Year 12 | Married | Out of work but not currently looking for work |
| ID-7 | Victoria | At least once a week | 3-5 years | 1 | Yes | Man | 65-74 | Australian | Some high school, but did not complete Year 12 | Married | Retired |
| ID-8 | Victoria (rural) | Daily |  | 3 | Yes | Woman | 55-64 | Australian | Completed more than 3-year university degree | Married | Self-employed |
| ID-9 | Victoria | Not at all but I have been a regular smoker | Within last year | 2 | Yes | Man | 45-54 | New Zealander | TAFE/diploma graduate | Defacto | Employed |
| ID-10 | Western Australia | Daily |  | 0 | Yes | Woman | 55-64 | New Zealander | TAFE/diploma graduate | Divorced | Employed |
| ID-11 | Queensland | Daily |  | 1 | Yes | Woman | 75-84 | New Zealander | Some high school, but did not complete Year 12 | Divorced | Retired |
| ID-12 | Queensland (rural) | Daily | 1-2 years | 3 | Yes | Woman | 55-64 | Australian | Completed a 3-year University degree | Defacto | Retired |
| ID-13 | New South Wales | Daily |  | 2 | Yes | Woman | 45-54 | Australian | Some high school, but did not complete Year 12 | Married | Stay at home |

* modified Medical Research Council Dyspnoea Scale
