# Supplementary material for: The primary care experience of adults with chronic obstructive pulmonary disease (COPD). An interpretative phenomenological inquiry
Source: PLoS One. 2023 Jun 23;18(6):e0287518. doi: 10.1371/journal.pone.0287518 (PMC10289323; doi:10.1371/journal.pone.0287518)
Supplement: S1 Fig — (PDF) [file pone.0287518.s003.pdf]

The primary care experience of adults with chronic obstructive pulmonary disease (COPD): Initial findings for participant feedback and comment

MONASH University

Introduction

Chronic Obstructive Pulmonary Disease (COPD) is a progressive respiratory illness and major source of morbidity and mortality across the world. Smoking is the principle risk factor for COPD. Qualitative studies describing the lived experience of COPD patients reveal a range of challenges during interactions with healthcare professionals that can be exacerbated by difficulties in quitting smoking. These studies have tended to take a narrow view of patient experience, or have not considered the stigmatising role of smoking in healthcare interactions.

The study explored the patient experience of Australian adults with COPD, many of who were current smokers. We focussed on experiences in Australian general practices were most of the care for mild-moderate COPD occurs.

Methods

- 13 participants were interviewed who responded to recruitment flyers posted on social media in COPD and carer support groups
- Interviews were held between February and June 2022 (phone and Zoom). Most participants were interviewed twice.
- Interviews used technique called semi-structured interviewing to explore different aspects of the experience of care.
- Interviews were audio recorded and transcribed verbatim so that they could be analysed.
- An analytic technique called Interpretive Phenomenological Analysis was used to understand the stories of participants and organise similar experiences into themes.

Key Characteristics of participants

		N = 13
Age	45-54	N=5
	55-64	N=5%
	65-74	N=2
	75+	N=1
Gender	Male	N=4
	Female	N=9
State	Victoria	N=7
	Queensland	N=4
	NSW	N=1
	Western Australia	N=1
Smoking status	Daily	N=7
	At least once a week	N=2
	Less than weekly	N=1
	Ex-smoker	N=3

Major Findings

An empathetic, pro-active and skilful GP with knowledge and interest in COPD is integral to good care experience

"…I have an actual great trust for my GP… [they are] awesome doctors … and they actually really do, like these guys once you go in they start to pump you through a system of okay, you haven't had this checked out and we've noticed that, so go and speak to this person here and they'll – do you know what I mean?" [ID-4, male, 45-55, Victoria]

Smoking created tensions in interactions with doctors

"It's always judged if you mention that you smoke. He hasn't brought it up for a couple of months because he knows I've been dealing with lots of major other things at the moment, but he does know I'm trying to give up again, and he always encourages me, and says to keep on trying, never quit quitting type thing." [ID-5, female, 45-54, Victoria]

Considerable work was required from patients to meet healthcare needs, gain knowledge and achieve personal empowerment and control

"So it's, it's just learning more about it and that's something that I never learnt from any GP. No GP has ever given me any written brochures, information, no one has ever said, look maybe you should go and see a specialist or anything. So it was just me asking…" [ID-6, female, 55-64, Queensland]

Judgement and stigma were powerful influences on care

"…you're … made to feel guilty because you're a smoker, I've done it to myself I know that. But I was (at) a point where I just wouldn't go to the doctors unless I was half dead because they made you feel so bad…" [ID-6, female, 55-64, Queensland]

COVID contributed further strain in healthcare interactions

"because you can't see the inside of me, and you can't – I don't have a sign on me – it was COVID – like I had a – I have a mask exemption because I can't wear a mask for any length of time. People, when I went anywhere … (some) would like, why aren't you wearing a mask… And so, that added more stigma to things, because you're not wearing a mask." ." [ID-11, female, 75-84, Queensland]

Pulmonary rehabilitation was a safe space and specialist care a knowledge bank

"And but then there were still people in my course (pulmonary rehabilitation course) that were still, still smoked. And stuff like that but they didn't, they didn't give them a hard time about it, and make them feel guilty" [ID-6, female, 55-64, Queensland]

Conclusions and Implications for practice

Participants described a range of experiences when interacting with health services for their COPD and several 'group experiential' themes were identified. Several participants felt their GP's were dismissive of their health concerns. They felt like a number and disrespected. These participants were less trusting of their GP's and made comments such as "they're only good for a prescription and a referral" [ID-1, female, 45-54, Victoria]. Physical difficulties including fatigue and breathlessness impacted experience of access, but so to did affective and relational factors. Stigma was a powerful underlying driver of care experience for this group that exhibited overt and covert impacts on help-seeking and access. Perceptions of medical and social vulnerability were magnified as a result of COVID19 and stigmatising experiences. Proactive, empathetic care was important to participants but this was not the experience of many participants. Many took on the 'work' of care themselves, which particularly impacted the experience of comprehensiveness and coordination of care.
